# The relationship of soluble urokinase plasminogen activator receptor levels with preterm premature rupture of membranes and predictive value in neonatal intensive care unit admission

**DOI:** 10.1590/1806-9282.20251351

**Published:** 2026-05-11

**Authors:** Bergen Laleli Koc, Atakan Tanacan, Ecem Haliloglu, Huriye Erbak Yilmaz, Duygu Tugrul Ersak, Betul Akgun Aktas, Ozgur Kara, Dilek Sahin

**Affiliations:** 1 Ankara Bilkent City Hospital, Department of Obstetrics and Gynecology, Division of Perinatology – Ankara, Turkey.; 2 University of Health Sciences, Ankara Bilkent City Hospital, Department of Obstetrics and Gynecology, Division of Perinatology – Ankara, Turkey.; 3 Ankara Bilkent City Hospital, Department of Obstetrics and Gynecology – Ankara, Turkey.; 4 İzmir Katip Celebi University, Department of Biochemistry – İzmir, Turkey.

**Keywords:** Inflammation, Pregnancy, Preterm premature rupture of the membranes, Receptors, urokinase plasminogen activator

## Abstract

**OBJECTIVE:**

Preterm premature rupture of membranes is a pregnancy complication in which infection and inflammation play an important role. Soluble urokinase plasminogen activator receptor has been described as a biomarker of infectious and inflammatory diseases. The aim of this study was to evaluate maternal serum soluble urokinase plasminogen activator receptor levels in pregnant women with preterm premature rupture of membranes and to determine the predictive value in neonatal outcomes.

**METHODS:**

In this prospective case-control study, complete blood count parameters, serum C-reactive protein, and soluble urokinase plasminogen activator receptor levels of 40 pregnant women with preterm premature rupture of membranes and 40 randomly selected healthy pregnant women between 22 and 34 weeks’ gestation were recorded. Neutrophil-to-lymphocyte ratio, systemic inflammation response index, and systemic immune-inflammation index were calculated. Receiver operating characteristic analysis assessed the predictive value of maternal serum soluble urokinase plasminogen activator receptor levels for neonatal intensive care unit admission.

**RESULTS:**

Maternal serum soluble urokinase plasminogen activator receptor levels, neutrophil-to-lymphocyte ratio, systemic immune-inflammation index, and systemic inflammation response index scores were significantly higher in the preterm premature rupture of membranes group than in controls (p<0.05). The soluble urokinase plasminogen activator receptor value in receiver operating characteristic analysis identified 4.33 ng/mL (62% sensitivity, 64% specificity) and 3.65 ng/mL (72% sensitivity, 73% specificity) for neonatal intensive care unit admission and adverse perinatal outcomes in the preterm premature rupture of membranes group (p<0.05).

**CONCLUSION:**

Soluble urokinase plasminogen activator receptor may be used as an ancillary marker in the prediction of neonatal intensive care unit admission and adverse perinatal outcome in preterm premature rupture of membranes cases, along with clinical findings.

## INTRODUCTION

Preterm premature rupture of membranes (PPROM) occurs when the fetal membranes rupture before the 37th week of pregnancy. PPROM affects 2–3% of pregnancies, while term PROM occurs in ∼8%^1,2^. It is a common cause of preterm labor, but its etiopathogenesis remains unclear. One of the factors in PPROM is oxidative stress (OS) and reactive oxygen species (ROS)^3^. Several risk factors, including infections, inflammation, local tissue hypoxia, urogenital infections, obesity, and smoking, contribute to PPROM cases before term^4^. PPROM increases maternal, fetal, and neonatal risks, including chorioamnionitis, sepsis, necrotizing enterocolitis, and respiratory distress syndrome (RDS)^5,6^.

Soluble urokinase plasminogen activator receptor (SuPAR) is the soluble form of the urokinase plasminogen activator receptor, a membrane protein anchored to glycosyl-phosphatidylinositol. Changes in SuPAR levels indicate inflammation and immune system activation. Serum SuPAR levels are used to measure the severity of inflammation and are considered a prognostic marker in patients with sepsis, systemic inflammation response index (SIRS), or bacteremia^7^. Unlike other inflammation markers, SuPAR is less affected by short-term changes. It helps predict mortality and morbidity, sharing similar risk factors with chronic systemic inflammation markers. Treatment and anti-inflammatory interventions can lower SuPAR levels^8^.

This study aimed to assess maternal serum SuPAR levels in pregnant women with PPROM and examine their role in predicting adverse perinatal outcomes.

## METHODS

### Type of study

This prospective case-control study was performed in the Ankara City Hospital Perinatology Department. The ethics committee approval was obtained with reference number E2-22-2390 and dated 14/09/2022. Informed consent was obtained from the participants.

### Study population

The minimum number of participants required for the study with 80% power was found to be 24 (12 for each group) according to the results of a recent study^9^. G Power software was used for sample size analysis. The study involved 40 pregnant women with PPROM and 40 healthy pregnant women without PPROM, aged similarly, and between 22 and 34 weeks of gestation. Amniotic fluid loss was confirmed either by vaginal examination with a sterile speculum for leakage or through the placental alpha microglobulin-1 (PAMG-1) test (AmniSure ROM Test). Women with multiple pregnancies or systemic or pregnancy-related diseases were excluded. None of the participants showed signs or symptoms of chorioamnionitis at admission or during follow-up. Controls were randomly selected from healthy women of similar maternal and gestational age.

Fetal measurements were taken, and the estimated fetal weight was calculated for all participants. The single-deepest pocket was measured to assess the fetal amniotic volume. Maternal and neonatal outcomes were extracted from hospital records.

### Data collection

Blood samples were collected from pregnant women diagnosed with PPROM when they were admitted to the perinatology clinic, before they received any medication or antibiotics. Hemogram results, neutrophil-to-lymphocyte ratio (NLR), and serum C-reactive protein (CRP) levels were recorded. The SIRI and systemic immune-inflammation index (SII) were also calculated.

In addition, serum SuPAR levels specific to this study were measured. Blood was drawn into tripotassium ethylenediaminetetraacetic acid tubes, then centrifuged at 3,600×*g* for 10 min to separate plasma. The plasma was stored at -80°C until analysis.

For SuPAR testing, plasma samples were added to enzyme-linked ımmunosorbent assay (ELISA) plate wells coated with antibodies. Samples were processed per kit protocol, and absorbance was read at 450 nm with a SuPAR ELISA kit and BioTek device (Elabscience, catalog no: E-EL-H2584, lot no: GNHXKBA3CK, ELx800, USA).

### Statistical analysis

Statistical analyses were performed using IBM SPSS Statistics version 25 (IBM Corp., Armonk, NY, 2017). As the data were normally distributed, group comparisons were conducted using the Student's t-test. Descriptive statistics were presented as means and standard deviations (SDs). Categorical variables were analyzed using the chi-square test and expressed as frequencies and percentages [n (%)]. A two-tailed p-value of <0.05 was considered statistically significant.

Pearson correlation was applied to evaluate the associations between neonatal intensive care unit (NICU) admission and maternal SuPAR levels, gestational age at delivery, CRP, SII, SIRI, and NLR in PPROM. A 5% Type I error level was used as the threshold for statistical significance. Furthermore, a correlation analysis was performed between SuPAR, body mass index (BMI), parity, and smoking status.

Perinatal complications such as sepsis, low birth weight (<1,500 g), and RDS were compared between the groups, and an receiver operating characteristic (ROC) analysis was performed to assess the utility of SuPAR in predicting adverse perinatal outcomes. Adverse neonatal outcome was defined as the presence of at least one of the following: neonatal sepsis, low birth weight (<1,500 g), and RDS.

To assess the predictive value of maternal serum SuPAR levels, SII, SIRI, and NLR for NICU admission, ROC curve analysis was performed. The optimal cutoff value was determined using the Youden index, with sensitivity and specificity reported. A 5% error rate was applied when evaluating area under the curve (AUC) values.

## RESULTS

Table 1 shows the clinical characteristics, perinatal outcomes, and maternal serum biomarkers for both the PPROM and control groups. The PPROM group had significantly lower gestational age at birth and neonate birth weight (p<0.001). BMI, NICU admission, and composite neonatal outcomes were higher in PPROM (p<0.001). Neonatal outcomes are summarized in Table 1. The 1- and 5-min Apgar scores of neonates also differed significantly between the groups (p=0.001, p=0.023). Although gestational age at sampling was matched, the groups were not matched on gestational age at birth. SuPAR level was higher in the PPROM group, 4.47±1.16 (mean±SD), than in the controls, 2.80±0.9 (mean±SD); p<0.001. Serum CRP, white blood cell (WBC), neutrophil, and monocyte counts also differed between the groups (p=0.002, p=0.001, p=0.001, p=0.03, respectively). The mean NLR was 6.15±2.94 in PPROM and 4.69±1.60 in the control group, with a statistically significant difference (p=0.008). SII and SIRI, indices of systemic inflammation, were higher in PPROM (p=0.001 and p=0.004, respectively). However, no correlation was found between SuPAR levels and other inflammation markers. No statistically significant correlations were observed among SuPAR, BMI, parity, and smoking (all p>0.13).

**Table 1:** Comparison of clinical, perinatal outcomes, and maternal serum biomarkers between preterm premature rupture of membranes and control groups.

Variable	PPROM group n: 40	Control group n: 40	p-value
Maternal age	28.42±5.89	27.10±5.21	0.290†
Gravidity	2.35±1.35	2.20±1.57	0.648†
Parity	0.87±1.11	0.72±0.87	0.505†
BMI	27.6±4.1	24.5±2.2	**<0.001** †
Gestational week, at blood sampling	28.5±4.0	27.4±2.9	0.134†
Gestational week, at birth	31.9±4.5	38.3±2.5	**<0.001** †
Birth weight	1,948±809	3,194±600	**<0.001** †
Smoking yes (%)/no (%)	3 (7.5%)/37 (92.5%)	0 (0%)/40 (100%)	0.07‡
Type of delivery VB (%)/CS (%)	18 (45%)/22 (55%)	16 (40%)/24 (60%)	0.651‡
NICU admission yes (%)/no (%)	29 (72.5%)/11 (27.5%)	5 (12.5%)/35 (87.5%)	**<0.001** ‡
Adverse neonatal outcome yes (%)/no (%)	28 (70%)/12 (30%)	3 (7.5%)/37 (92.5%)	**<0.001** ‡
APGAR 1 <7/≥7	17 (42.5%)/23 (57.5%)	4 (10%)/36 (90%)	**0.001** ‡
APGAR 5 <7/≥7	9 (22.5%)/31 (77.5%)	2 (5%)/38 (95%)	**0.023** ‡
Sepsis yes (%)/no (%)	5 (12.5%)/35 (87.5%)	0 (0%)/40 (100%)	**0.021** ‡
Respiratory distress syndrome yes (%)/no (%)	21 (52.5%)/19 (47.5%)	0 (0%)/40 (100%)	**<0.001** ‡
Low birth weight (<1,500 g) yes (%)/no (%)	10 (25%)/30 (75%)	1 (2.5%)/39 (97.5%)	**0.003** ‡
SuPAR (ng/mL)	4.47±1.16	2.80±0.9	**<0.001** †
CRP (mg/dL)	10.31±15.84	2.39±1.70	**0.002** †
WBC (×109/L)	11.62±3.21	9.57±2.06	**0.001** †
Neutrophil (×109/L)	9.19±3.07	7.21±1.86	**0.001** †
Monocyte (×109/L)	0.56±0.23	0.46±0.12	**0.030** †
Lymphocyte (×109/L)	1.66±0.56	1.63±0.42	0.752†
Platelet (×109/L)	260±75	233±47	0.060†
NLR	6.15±2.94	4.69±1.60	**0.008** †
SII	1,554±784	1,085±410	**0.001** †
SIRI	3.35±2.11	2.23±1.07	**0.004** †

† Student's t-test; results were presented as mean±SD.

‡ Chi-square test; results were presented as number (%).

p<0.05 values were presented in bold. PPROM: preterm premature rupture of the membranes; BMI: body mass index; NICU: neonatal intensive care unit; SuPAR: soluble form of the urokinase-type plasminogen activator receptor; WBC: white blood count; NLR: neutrophil/lymphocyte ratio; CRP: C-reactive protein; SII: systemic immune inflammation index; SIRI: systemic inflammation response index; VB: vaginal birth; CS: cesarean section.

Pearson correlation in Table 2 showed a significant positive correlation between SuPAR and NICU admission in PPROM (p=0.039). There was also a significant negative correlation between NICU admission and gestational age at delivery (p=0.004). Other markers were not significantly correlated with NICU admission.

**Table 2:** Correlation analyses between neonatal intensive care unit admission, soluble urokinase plasminogen activator receptor, gestational age at delivery, C-reactive protein, systemic immune inflammation index, systemic inflammation response index, and neutrophil/lymphocyte ratio in the preterm premature rupture of membranes group.

Variable		Gestational week, at birth	SuPAR	CRP	SII	SIRI	NLR
NICU admission	Pearson correlation	-0.441	0.328	-0.006	-0.058	-0.087	0.018
	p-value	**0.004**	**0.039**	0.970	0.721	0.594	0.914
	N	40	40	40	40	40	40

Pearson correlation test. p<0.05 values were presented in bold. SuPAR: soluble urokinase plasminogen activator receptor; CRP: C-reactive protein; SII: systemic immune-inflammation index; SIRI: systemic inflammation response index; NLR: neutrophil-to-lymphocyte ratio; NICU: neonatal intensive care unit.

**Figure 1:**
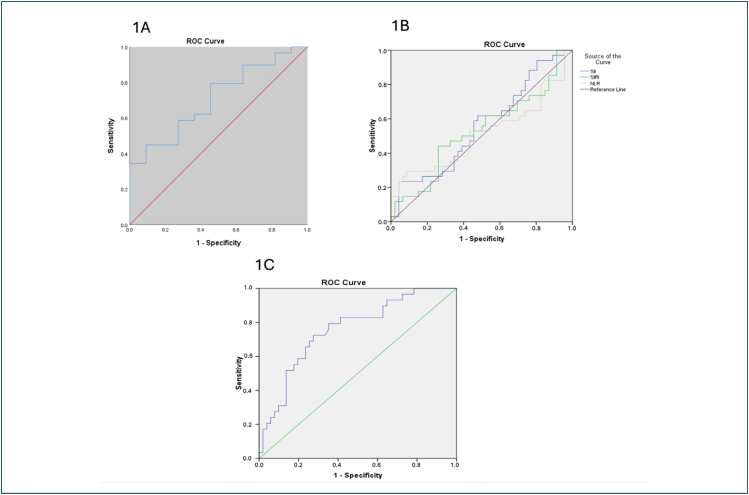
(A) Receiver operating characteristic curve for soluble urokinase plasminogen activator receptor in predicting neonatal intensive care unit admission in the preterm premature rupture of membranes group. (B) Receiver operating characteristic analysis for other systemic inflammation indices in predicting neonatal intensive care unit admission. (C) Receiver operating characteristic curve for soluble urokinase plasminogen activator receptor in predicting composite adverse perinatal outcome.

Figure 1A presents the ROC curve for SuPAR's effectiveness in predicting NICU admission. The AUC was 0.708 (95%CI 0.53–0.87, p=0.04) for SuPAR. The best balance of sensitivity and specificity in the ROC curve was found at 4.33 ng/mL, with 62.1% sensitivity and 63.6% specificity for NICU admission. Figure 1B presents the ROC analysis for other systemic inflammation indices’ utility in predicting NICU admission. None of them had significant potential for clinical usage (AUC≤0.55, p>0.05; Figure 1B).

For predicting adverse perinatal outcome, the optimal SuPAR cutoff was 3.65 ng/mL (72% sensitivity, 73% specificity; AUC=0.75, 95%CI 0.64–0.86, p<0.001; Figure 1C).

## DISCUSSION

This study revealed that pregnancies complicated with PPROM had higher SuPAR levels compared to the low-risk controls. Moreover, SuPAR had potential as a biomarker to predict NICU admission in PPROM cases but the predictive value of gestational age at birth was greater than that of SuPAR. Although CRP, NLR, SII, and SIRI were higher in PPROM, none correlated with SuPAR. Thus, SuPAR may serve as a potential biomarker to predict NICU admission in pregnant women with PPROM. SuPAR outperformed other markers but remained inferior to gestational age, showing limited utility due to modest sensitivity and specificity.

SuPAR levels increase with the activation of the immune system by various infections. SuPAR increases in cardiovascular, rheumatologic, metabolic, and infectious diseases^10-13^. In a study involving 273 intensive care patients, SuPAR was higher in intensive care patients, especially with sepsis. In this study, CRP and procalcitonin (PCT) were found to have a greater diagnostic value than SuPAR in sepsis^14^. Similarly, in another study, SuPAR levels were found to be significantly higher in patients with bacterial infection, but SuPAR was not diagnostically superior to CRP and PCT in detecting bacterial growth in blood culture^15^. In a study conducted on patients suffering from acute exacerbations of chronic obstructive pulmonary disease, SuPAR outperformed CRP and fibrinogen in identifying acute exacerbations of chronic obstructive pulmonary disease^16^.

Serum SuPAR levels have been studied in obstetrics, particularly in relation to preeclampsia. One study comparing third-trimester preeclamptic pregnancies with healthy controls reported elevated plasma SuPAR levels in the preeclamptic group. IL-6 and CRP were also elevated^17^. However, a 2023 cohort study examined changes in SuPAR levels throughout pregnancy and their association with hypertensive disorders. The study reported median SuPAR levels after 24 weeks’ gestation as 2.43 (interquartile range [IQR]=0.91) in women with hypertensive disorders and 2.12 (IQR=0.79) in those without (p=0.11), concluding that SuPAR levels did not significantly differ between the two groups at any stage of pregnancy^18^.

To our knowledge, only one study has examined SuPAR levels in PPROM cases. In this study, 49 PPROM cases between 24 and 34 weeks of gestation were analyzed, and patients were divided into subgroups with and without histological chorioamnionitis. The study found that uPAR, with a cutoff of 6.4 ng/mL, better predicted infection in PPROM than CRP and total leukocyte count, showing higher sensitivity and specificity. The study concluded that these biomarkers can accurately detect infection, even without clinical symptoms^19^.

SuPAR's strong association with infection and the immune response makes it valuable for clinical use in PPROM cases. We believe it provides more reliable information about adverse neonatal outcomes compared to traditional inflammatory markers. The fetus responds to maternal infection and inflammation through cytokines, leading to "fetal inflammatory response syndrome"^20^. Maternal inflammation likely influences fetal outcomes, and sensitive markers may help predict morbidity.

The main limitation of this study is the small sample size. Another limitation is that patients with suspected chorioamnionitis were not included. The groups were not matched based on gestational age at birth, which may affect the interpretation of the outcome differences. However, blood samples for SuPAR levels were taken from participants at similar gestational ages.

## CONCLUSION

Previous studies suggest that SuPAR could be an early marker for infections and inflammation, and it may serve as both a diagnostic and prognostic biomarker. Adverse perinatal outcomes are closely related to the severity of infection/inflammation and the gestational age at delivery in PPROM cases. While gestational age is the main factor for NICU admission, inflammatory markers also help predict NICU admission. SuPAR may serve as an ancillary marker for predicting NICU admission and adverse outcomes in PPROM, alongside clinical findings.

## Data Availability

The datasets generated and/or analyzed during the current study are available from the corresponding author upon reasonable request.
